# Enhanced Fear Memory in Adult Male C57BL/6 Mice Following Low‐Dose Isoflurane Exposure During Juvenile Development

**DOI:** 10.1002/brb3.70816

**Published:** 2025-09-30

**Authors:** Jing Sun, Yong Tang, Jinlong Chang, Jianbang Lin, Li Luo, Yuantao Li, Guanxiong Wu, Yuting Hu, Zhao Zheng, Ye Zhang

**Affiliations:** ^1^ Department of Anesthesiology Shenzhen Futian District Maternal and Child Health Hospital Shenzhen China; ^2^ Shenzhen Maternity and Child Healthcare Hospital, Women and Children's Medical Center Southern Medical University Shenzhen China; ^3^ Brain Cognition and Brain Disease Institute, Shenzhen Institutes of Advanced Technology Chinese Academy of Sciences Shenzhen China; ^4^ Department of Physiology and Pathophysiology, School of Basic Medical Sciences Dali University Dali China; ^5^ Shenzhen Technological Research Center for Primate Translational Medicine, Shenzhen Key Laboratory for Molecular Biology of Neural Development, Shenzhen‐Hong Kong Institute of Brain Science, Shenzhen Institute of Advanced Technology Chinese Academy of Sciences Shenzhen China; ^6^ University of Chinese Academy of Sciences Beijing China; ^7^ Biomedical Research Institute Hubei University of Medicine Shiyan China; ^8^ Department of Traditional Chinese Medicine Shenzhen Futian District Maternal and Child Health Hospital Shenzhen China

**Keywords:** fear, isoflurane, safety assessment, sedation, slow gamma oscillations, spatial cognition

## Abstract

**Objective:**

To elucidate the neural mechanisms through which neonatal exposure to low‐dose anesthetic isoflurane (ISO) enhances adult fear memory, specifically examining neural oscillations and activation patterns in the mediodorsal thalamus (MDL), dentate gyrus (DG), anterior cingulate cortex (ACC), and medial entorhinal cortex (MEC).

**Methods:**

Male C57BL/6 mice received 0.75% ISO or saline injections at postnatal day 7 (P7). At 6 months, contextual fear‐memory testing was conducted (Saline + Air: *n* = 14; Saline + ISO: *n* = 13). Neural correlates were assessed via in vivo electrophysiology during behavioral tasks and c‐Fos immunohistochemistry in the MDL, DG, ACC, and MEC.

**Results:**

ISO‐exposed mice exhibited significantly enhanced fear memory, with >75% displaying freezing as the dominant conditional response (vs. controls). This was accompanied by increased c‐Fos+ cell density in the MDL and altered slow gamma oscillation power in fear‐processing circuits. Non‐freezing responses (e.g., jumping/darting) were reduced in the Saline + ISO group.

**Interpretation:**

Early low‐dose ISO intervention in neonatal mice enhances adult fear memory, potentially through MDL activation and slow gamma oscillation modulation. These findings indicate that low‐dose ISO may improve cognitive function by optimizing fear memory consolidation, contrasting with known detrimental effects of high‐dose anesthetics on neurodevelopment.

## Graphical abstract

Adult male C57BL/6 mice exhibit enhanced fear memory after exposure to low doses of isoflurane during juvenile development.

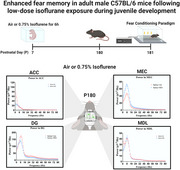

## 1 Introduction

Preclinical studies have indicated that exposure to isoflurane (ISO) during early life stages may have implications for neurocognitive development. In a study carried out by Loepke et al. (2009) on 7‐day‐old mice exposed to 6 h of 1.5% ISO, no effects were observed on adult spontaneous motor activity or on spatial learning and memory performance, despite an increase in plasma S100 and neonatal cell loss. Moreover, Yi et al. (2015) reported that treatment of 7‐day‐old mice with 0.75% ISO for 6 h resulted in increased neurodegenerative biomarker S100β in the blood, yet no significant alterations in memory and learning ability were observed. Contrastingly, studies by Yi et al. (2015), Liu et al. (2017), and Sen and Sen (2016) involving mice exposed to ISO at the age of 7 days demonstrated heightened expression of caspase‐3, prolonged escape latency, and impaired transcriptional activity of cyclic AMP response element‐binding (CREB). Schaefer et al. (2020) found that postnatal day 7 mice exposed to 1.5% ISO for 4 h exhibited decreased mushroom spines and deficits in object recognition and fear memory. On the basis of the above literatures, the effect of exposure to 1.5% ISO on cognitive performance at P7 mice is controversial. ISO is a sedative that is frequently used in the examination and treatment of young children due to its use in clinical situations. Therefore, to determine the long‐term effects of ISO on cognitive performance, we performed behavioral, immunofluorescent staining techniques, and in vivo electrophysiological experiments related to fear memory in male adult mice that experienced 6 h of stimulation with 0.75% ISO (0.57 minimum alveolar concentration [MAC], the sedative dosage) at a juvenile stage.

To delve into the neurocircuit mechanisms underlying the impact of ISO on long‐term cognitive function in young mice, we conducted in vivo electrophysiological experiments to probe the effects of ISO on neuronal oscillations across various brain regions. The cognitive circuits within the brain are intricate and multifaceted, with pivotal regions playing essential roles in processing and regulating information. The medial entorhinal cortex (MEC), renowned for its reception of inputs from distinct cortical hierarchy streams, particularly the “where” pathways, acts as a conduit for transmitting neuronal signals to the hippocampus (Fernandez‐Ruiz et al. 2021; Sasaki et al. 2015). Additionally, the anterior cingulate cortex (ACC), implicated in fear behavior, exerts its modulatory influence on vicarious freezing behavior through projections to the lateral portion of the mediodorsal thalamus (MDL) (Kim et al. 2012; Fernandez‐Ruiz et al. 2021). The ACC's involvemen in fear‐related responses underscores its pivotal role within the neural network governing emotional reactions.

Immediate‐early genes, such as c‐Fos, are swiftly and transiently expressed in the brain in response to various stimuli (Robertson 1992; Wang et al. 2021). Notably, fear memory is linked to a c‐Fos activation map, reflecting the dynamic neuronal responses involved in the encoding and retrieval of fear‐related information (Roy et al. 2022; Silva et al. 2019). In our investigation, brain slices obtained 90 min after the completion of the memory recall test underwent immunohistochemical staining for c‐Fos. Intriguingly, a notable disparity in c‐Fos density was observed in the MDL between the ISO‐exposed group and the control group. Conversely, other regions of interest exhibited no discernible differences, indicating a specific impact on MDL function. Crucially, our findings suggest that the ability of the “where” pathways to engage in information exchange remains unaffected by ISO exposure. Furthermore, the ACC, a pivotal player in modulating fear behavior, remained unaltered following exposure to the sedative dose of ISO. These insights elucidate the nuanced effects of ISO on cognitive circuitry, underscoring the specificity of its influence on fear‐related memory processing and the modulation of freezing behavior via the ACC‐MDL pathway.

Neural oscillations, stemming from the coordinated activity of local neuron populations or assemblies across brain regions, play a pivotal role in encoding functional information. These rhythmic fluctuations, detectable through local field potential (LFP), provide insights into the functional dynamics of diverse brain areas. Our study delves into the impact of neonatal exposure to 0.75% ISO on LFP oscillations across various frequency bands.

Theta (4–12 Hz) and gamma (30–100 Hz) oscillations are commonly observed in the hippocampus during contextual exploration (Cardoso‐Cruz et al. 2013). Theta and gamma oscillations facilitate communication between the MEC and dentate gyrus (DG) (Fernandez‐Ruiz et al. 2021; Stepan et al. 2012). Additionally, theta and beta (15–30 Hz) oscillations in the DG encode encounters with cues (Rangel et al. 2015), whereas delta (1–4 Hz), alpha (8.5–12 Hz), and gamma oscillations in the ACC encode freezing responses (Kim et al. 2014). Theta oscillations in the MDL are heightened in chronic pain states (Cardoso‐Cruz et al. 2013). Further granularity emerges in gamma rhythms, classified into low gamma (25–40 Hz), medium gamma (40–65 Hz), and high gamma (65–85 Hz), each reflecting distinct neural dynamics in processing spatial frequency information in the visual system (Han et al. 2021).

Our findings unveil significant differences across entorhinal cortex bands, suggesting that neonatal ISO sedation disrupts spatial information processing in mice. Furthermore, the hippocampal capacity to operate through beta and low gamma oscillations is impacted, highlighting a broader influence on cognitive processes. Moreover, gamma oscillations in the ACC are implicated in reinforcing fear messages.

In summary, our study illustrates that extended neonatal exposure to ISO sedation leads to mild cognitive impairment in adulthood. This impairment is associated with disturbances in acquiring environmental information and assessing safety, highlighting the intricate interaction of neural oscillations in cognitive function. Our experiments offer novel insights into the potential cognitive ramifications of prolonged ISO exposure during early life.

## 2 Materials and Methods

### 2.1 Animal Housing and Care

Neonatal mice were reared by their mothers, and upon weaning, they were group‐housed in cages with ad libitum access to food and water. Male mice at 7 days postnatal age used in this experiment were used for the study of the improvement of ISO at early low doses. The animal housing facility maintained a temperature of 25°C ± 2°C and operated on a 12/12‐h light/dark cycle.

### 2.2 Neonatal ISO Exposure

All mice were randomly assigned to one of two groups: the control group and the ISO group. On postnatal day (PND) 7, mice in both groups received an injection of 3 mL/kg saline. After a 20‐min interval, the control group was exposed to air for 6 h at 37°C, whereas the ISO group underwent exposure to 0.75% ISO for the same duration at the same temperature. Following the anesthesia period, all mice were placed in a new home cage maintained at 37°C for 30 min before being returned to their respective parent cage.

### 2.3 Fear Conditioning Paradigm

Starting from postnatal day 180, mice underwent contextual fear conditioning (CFC). Behavioral tests were conducted between 08:00 and 18:00 in a room with low lighting (approximately 25 lx) and a controlled temperature of 22°C ± 2°C. Mice were individually placed in a circular shock chamber (Φ35 cm) with white walls featuring dark stick figures. The chamber's bottom consisted of metal buttons capable of delivering foot shocks. The chamber was scented with 25% alcohol. The conditioning protocol included a 180 s exploration period, followed by five foot shocks (0.4 mA, 1 s duration) administered at 180, 270, 370, 480, and 700 s, with an 80‐s post‐shock period. Natural memory recall tests (3 min) were conducted 1 day later. The entire training session for each mouse was recorded using a high‐resolution camera positioned atop the enclosure. EthoVision XT (Noldus Information Technologies) software was employed to automatically determine and record freezing behavior on the basis of mobility scores. Mice were categorized into freezing state (mobility score <1.3%), normal state (mobility score >1.3% and <45%), or mania state (mobility score >45%).

### 2.4 Immunofluorescence

Six mice were randomly chosen from each experimental group for immunohistochemistry. Approximately 90 min after the completion of the memory recall test, the mice were deeply anesthetized with an intraperitoneal injection of sodium pentobarbital and transcardially perfused first with ice‐cold phosphate‐buffered saline (PBS) followed by 4% paraformaldehyde (PFA). The brains were fixed in 4% PFA at 4°C for 24 h, followed by immersion in 20% sucrose in PBS at 4°C for 48 h and subsequent immersion in 30% sucrose in PBS at 4°C for another 48 h for cryoprotection. The cryosectioned brain tissues were stored at 4°C in 30% sucrose. Brain tissue sections, cut into 30‐µm‐thick slices, underwent immunofluorescence with the following steps: incubation with 10% serum at room temperature for 2 h, overnight incubation with primary antibodies against c‐Fos [#2250] (Cell Signaling Technology, Danvers, MA, USA) at 4°C, two 5‐min PBS washes, 3‐h incubation with secondary antibodies (ab150077) at room temperature, two 5‐min PBS washes, 5‐min 4′,6‐diamidino‐2‐phenylindole (DAPI) staining, and finally, two 5‐min PBS washes. The expression levels of c‐Fos were determined by three unbiased professionals who counted the numbers of c‐Fos‐positive cells.

### 2.5 LFP Electrophysiology

Mice were rendered unconscious through anesthesia, allowing for the exposure of the skull to facilitate electrode implantation. Precisely drilled burr holes were used to position nichrome wire electrodes, each with a diameter of 65 µm and provided by A‐M System, Inc., into targeted brain regions: the right ACC at Anterior–Posterior (AP): +1.0 mm, Medial–Lateral (ML): 0.3 mm, Dorsal–Ventral (DV): 1.2 mm; the MDL at AP: −1.0 mm, ML: 0.5 mm, DV: 3.2 mm; the DG at AP: −1.67 mm, ML: 1.0 mm, DV: 2.2 mm; and the MEC at AP: −5.0 mm, ML: 3.1 mm, DV: 3.5 mm. To establish a reference point and ground connection, supplementary screws were embedded in the contralateral frontal area and the cerebellar region. The electrode leads were then integrated into a 7‐pin socket, which served as the link to the Neurologger system. Dental cement was applied to firmly fix the electrodes and the socket in position.

After a week of recovery post‐surgical procedure, the mice were gradually introduced to a Neurologger dummy device for a period of 3 days to acclimate. On the day of the experiment, the Neurologger system was engaged, and LFPs were initially captured in the home cage for a 5‐min duration to establish a baseline, utilizing a sampling rate of 1600 Hz. Following this, the mice were relocated to a fresh home cage environment. Approximately 35 min later, LFPs were rerecorded for a 3‐min period within a circular shock chamber as part of the memory recall tests. The collected LFP data were then subjected to analysis using MATLAB software.

To optimize signal clarity and reduce noise, a notch filter was implemented to eliminate the 50 Hz power line frequency, and the data signals were detrended. For a detailed power spectrum analysis, the Chronux toolbox was utilized with settings configured to a time window (TW) of 3 s and a kernel (*K*) value of 5. The mean power across various frequency bands was computed to provide an in‐depth analysis of the neural activity.

### 2.6 Statistical Analysis

All data are presented as mean ± standard error of the mean. Within‐group comparisons were conducted using Student's *t*‐test. Significance was determined at *p < 0.05.

## 3 Results

### 3.1 Neonatal Exposure to 0.75% ISO Increases Fear Memory in Adult Wild‐Type Mice

**FIGURE 1:**
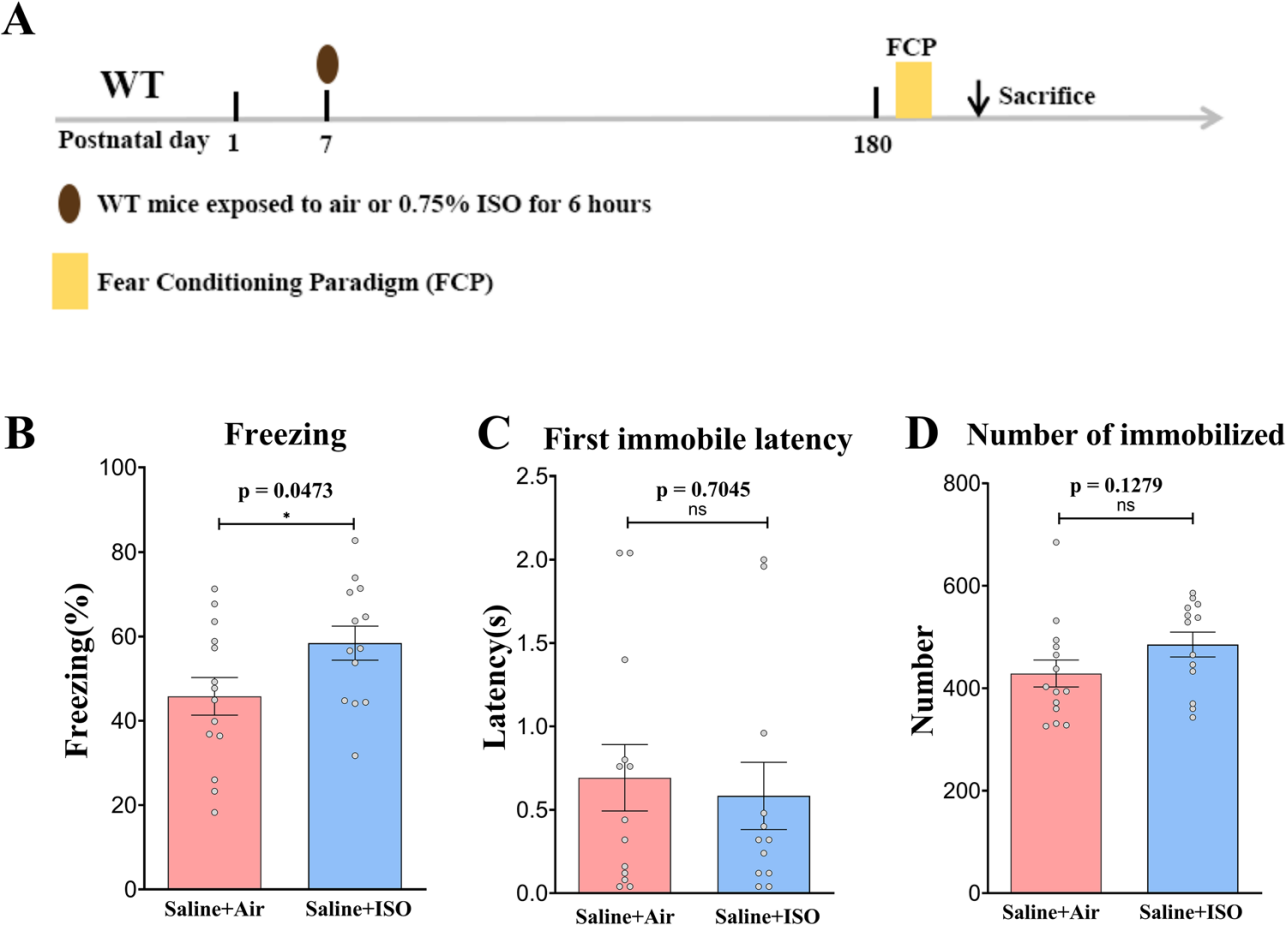
**Neonatal exposure to 0.75% isoflurane increases freezing behavior in adult wild‐type mice**. (A) Timeline of experimental procedures in WT mice. (B) In the conditioned fear test, the percentage cumulative duration of freezing in the saline + ISO mice was significantly higher than that in the saline + air mice. Data are presented as mean ± SEM. *n* = 13–14 mice/group. *p < 0.05. (C) In the conditioned fear test, there is no difference in the latency of first freezing behavior between mice in the saline + ISO group and saline + air group. Data are presented as mean ± SEM. *n* = 13–14 mice/group. ns, not significant. (D) In the conditioned fear test, the frequency of freezing behavior in the saline + ISO mice does not differ from that of mice in the saline + air group. Data are presented as mean ± SEM. *n* = 13–14 mice/group. ns, not significant.

To investigate the cognitive impact of 0.75% ISO exposure on adult mice aged 6–8 months, administered on postnatal day 7, we utilized a fear conditioning paradigm to assess safety assessment (Figure 1A). The cumulative freezing duration in mice from the ISO group was significantly prolonged compared to the control group (Saline + Air group: 45.8 ± 4.46, *n* = 14; Saline + ISO group: 58.4 ± 4.03, *n* = 13; unpaired *t* test, *t*(25) = 2.086, *p* = 0.0473, Figure 1B). However, freezing latency to the first freeze and freezing frequency did not exhibit significant differences between the Saline + Air and Saline + ISO groups (Figure 1C, unpaired *t* test, *t*(23) = 0.3840, *p* = 0.7045; Figure 1D, unpaired *t* test, *t*(25) = 1.575, *p* = 0.1279).

### 3.2 Neonatal 0.75% ISO Exposure Increases c‐Fos Expression in Fear Memory Circuits

**FIGURE 2:**
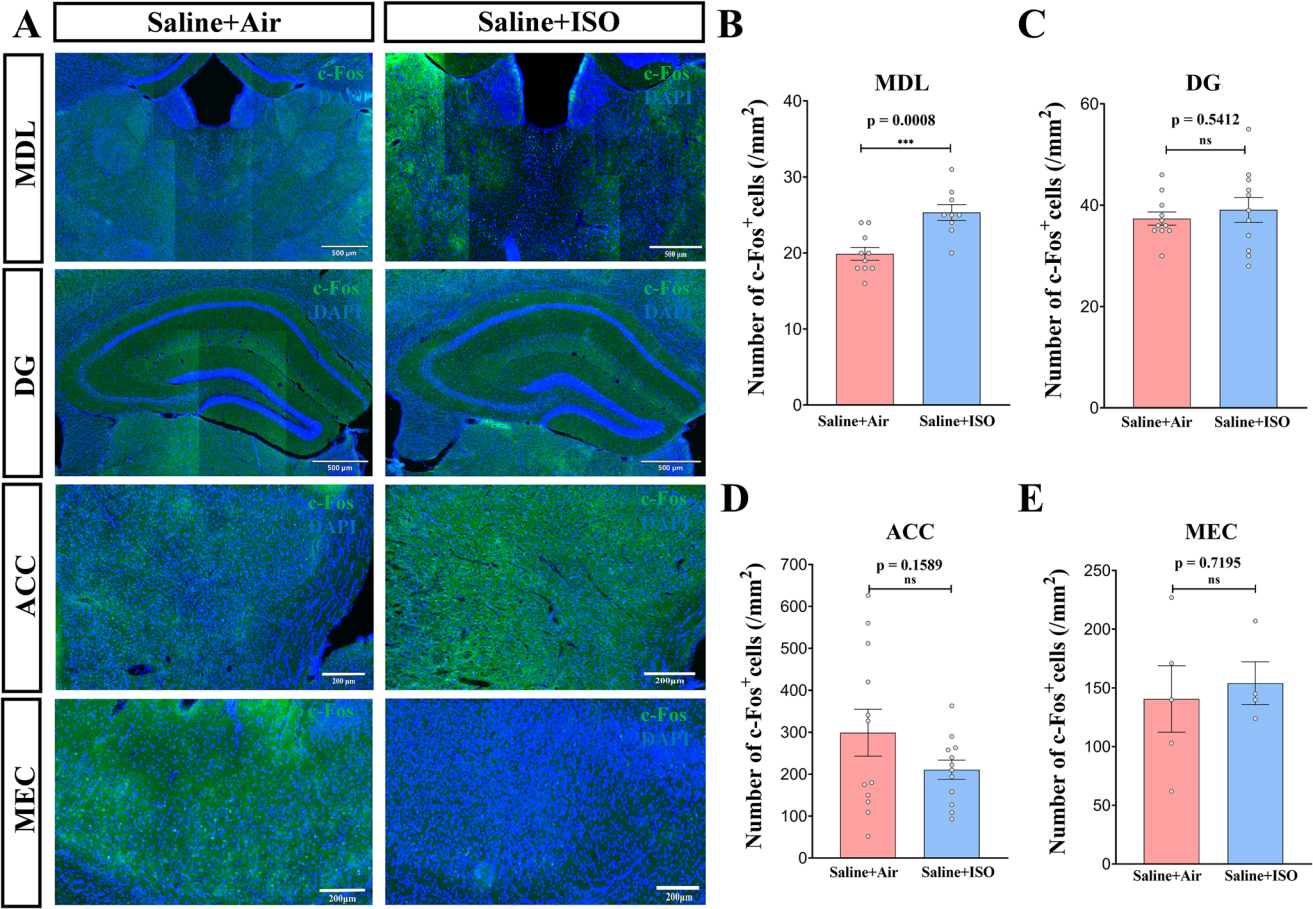
**Neonatal exposure to 0.75% isoflurane significantly increases the number of c‐Fos‐positive cells in the medial thalamus of wild‐type mice in the memory recall test**. (A) Immunofluorescent staining of c‐Fos (green) and DAPI (blue) in four brain regions (including medial thalamus, dentate gyrus [DG], anterior cingulate cortex [ACC], and medial entorhinal cortex [MEC]) of mice 90 min after memory recall test. MDL and DG, scale bar = 500 µm; ACC and MEC, scale bar = 200 µm. (B) The number of c‐Fos‐positive cells in the mediodorsal thalamus (MDL) of wild‐type mice in the saline + ISO group is significantly increased compared with those in the saline + air group. Data are presented as mean ± SEM. *n* = 9–10 mice/group. ***p < 0.001. (C) There is no significant difference in the number of c‐Fos‐positive cells in the DG of wild‐type mice in the saline + ISO group compared to the saline + air group. Data are presented as mean ± SEM. *n* = 11 mice/group. ns, not significant. (D) There is no significant difference in the number of c‐Fos‐positive cells in the ACC of wild‐type mice in the saline + ISO group compared to the saline + air group. Data are presented as mean ± SEM. *n* = 12 mice/group. ns, not significant. (E) There is no significant difference in the number of c‐Fos‐positive cells in the MEC of wild‐type mice in the saline + ISO group compared to the saline + air group. Data are presented as mean ± SEM. *n* = 4–5 mice/group. ns, not significant.

To investigate the effects of ISO on spatial cognition and safety assessment circuits in adult mice, we conducted fluorescent immunostaining for c‐Fos in the MEC, DG, ACC, and MDL following the memory recall test at 90 min (Figure 2A–E; both hemispheres/mice, six mice/group). The numbers of c‐Fos‐positive neurons were similar in the DG, ACC, and MEC between the two groups (Figure 2C, unpaired *t* test, *t*(20) = 0.6216, *p* = 0.5412; Figure 2D, unpaired *t* test, *t*(22) = 1.458, *p* = 0.1589; Figure 2E, unpaired *t* test, *t*(7) = 0.3740, *p* = 0.7195). However, the density of c‐Fos‐positive cells of MDL in the Saline + ISO group was significantly higher than that in the Saline + Air group (Saline + Air group: 19.9 ± 0.84, *n* = 10; Saline + ISO group: 25.33 ± 1.04, *n* = 9; Figure 2B, unpaired *t* test, *t*(17) = 4.079, *p* = 0.0008).

### 3.3 Neonatal 0.75% ISO Exposure Induces Abnormal LFPs Activity in Adult Mice

**FIGURE 3:**
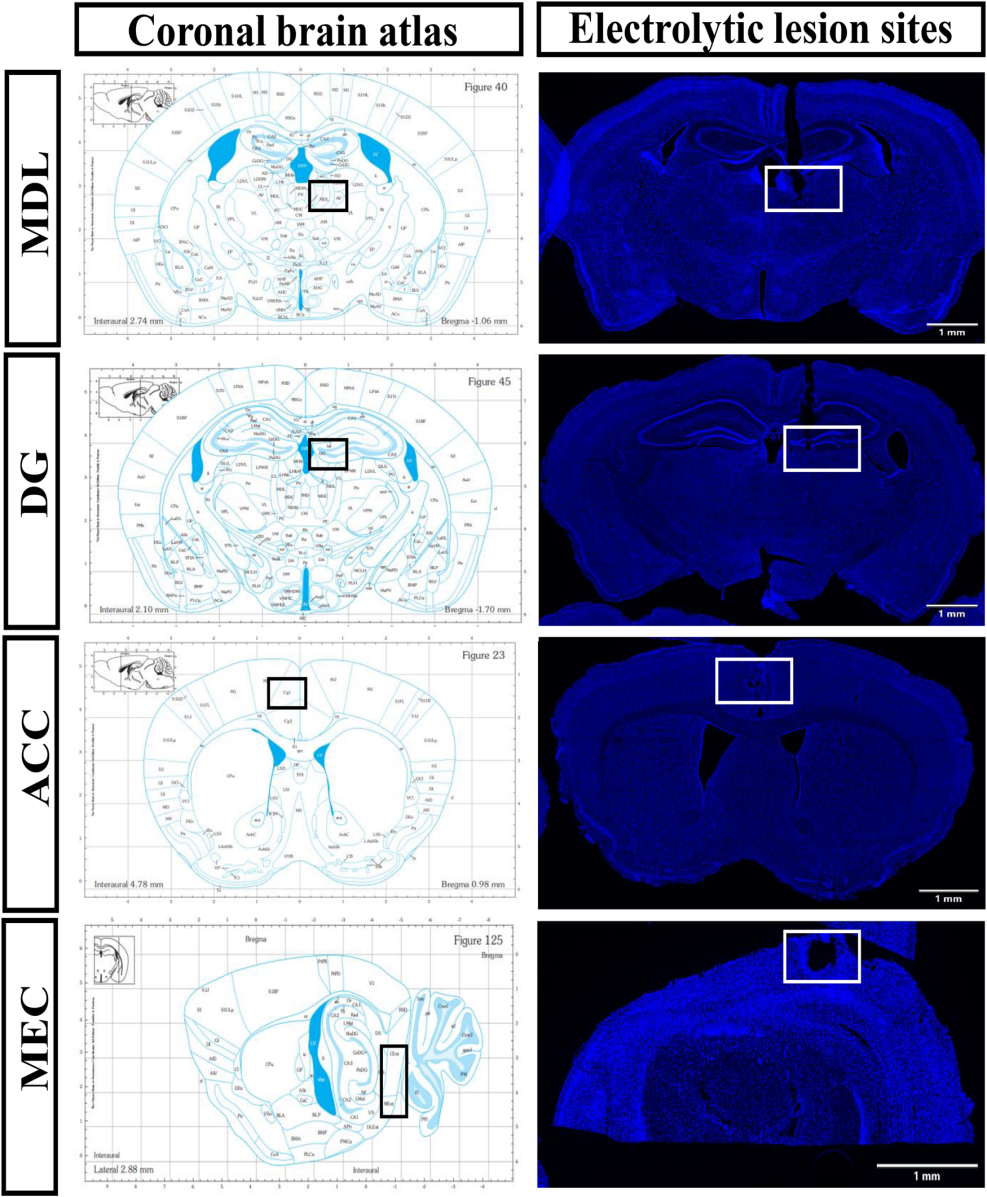
**Location of in vivo recording electrodes**. Two‐dimensional coronal reference atlas of mediodorsal thalamus (MDL), dentate gyrus (DG), anterior cingulate cortex (ACC), and medial entorhinal cortex (MEC). Representative DAPI‐stained brain sections show electrolytic damage produced by LFP electrodes placed in MEC, DG, ACC, and MDL. Scale bar = 1 mm.

During the memory recall tests phase, LFP were recorded in the MEC, DG, ACC, and lateral part of the MDL. DAPI‐stained brain sections unveiled electrolytic lesions (Figure 3A–D).

### 3.4 Medial Entorhinal Cortex

**FIGURE 4:**
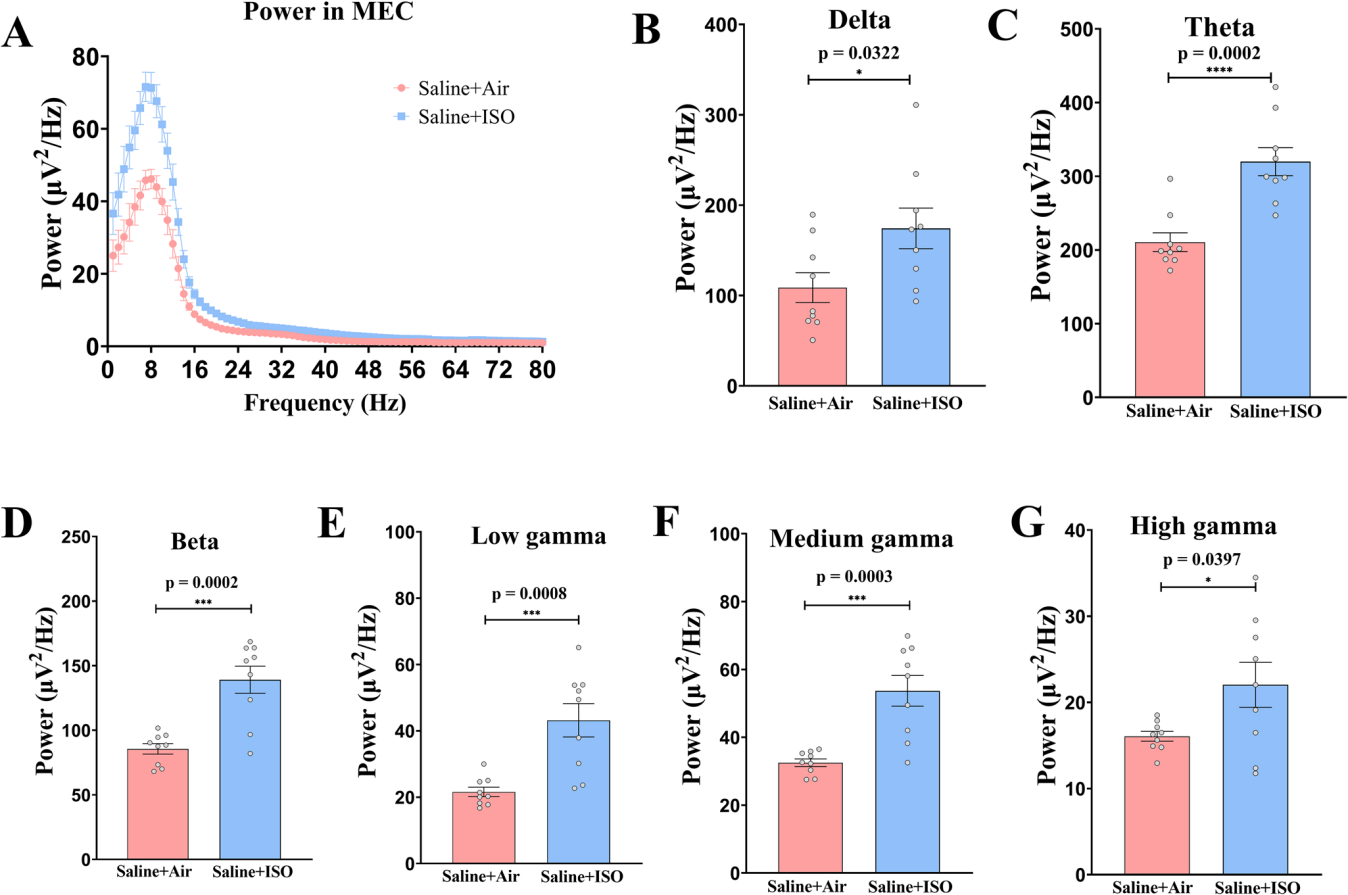
**Average power spectra of the medial entorhinal cortex of mice in the saline + air and saline + ISO groups differs significantly**. (A) Average power spectra obtained for local field potential (LFP) recording in the medial entorhinal cortex (three fragments/mice, three mice/group) during memory recall test. (B–G) Average power spectra in the delta (1–4 Hz; B), theta (4–12 Hz; C), beta (15–25 Hz; D), low gamma (26–40 Hz; E), medium gamma (41–65 Hz; F), and high gamma (66–80 Hz; G) bands, which implies that saline + ISO group mice have higher energy in different frequency energy spectra in the MEC brain region. *p < 0.05; ***p < 0.001; ****p < 0.0001. Data are presented as mean ± SEM.

The average power spectra exhibited differences in the MEC between the Saline + Air and Saline + ISO groups (Figure 4A). Particularly noteworthy was the increased mean power observed in the Saline + ISO group compared to the Saline + Air group across the delta (Figure 4B, unpaired *t* test, *t*(16) = 2.346, *p* = 0.0322), theta (Figure 4C, unpaired *t* test, *t*(16) = 4.772, *p* = 0.0002), beta (Figure 4D, unpaired *t* test, *t*(16) = 4.762, *p* = 0.0002), low gamma (Figure 4E, unpaired *t* test, *t*(16) = 4.132, *p* = 0.0008), medium gamma (Figure 4F, unpaired *t* test, *t*(16) = 4.540, *p* = 0.0003), and high gamma (Figure 4G, unpaired *t* test, *t*(16) = 2.240, *p* = 0.0397) bands.

### 3.5 Dentate Gyrus

**FIGURE 5:**
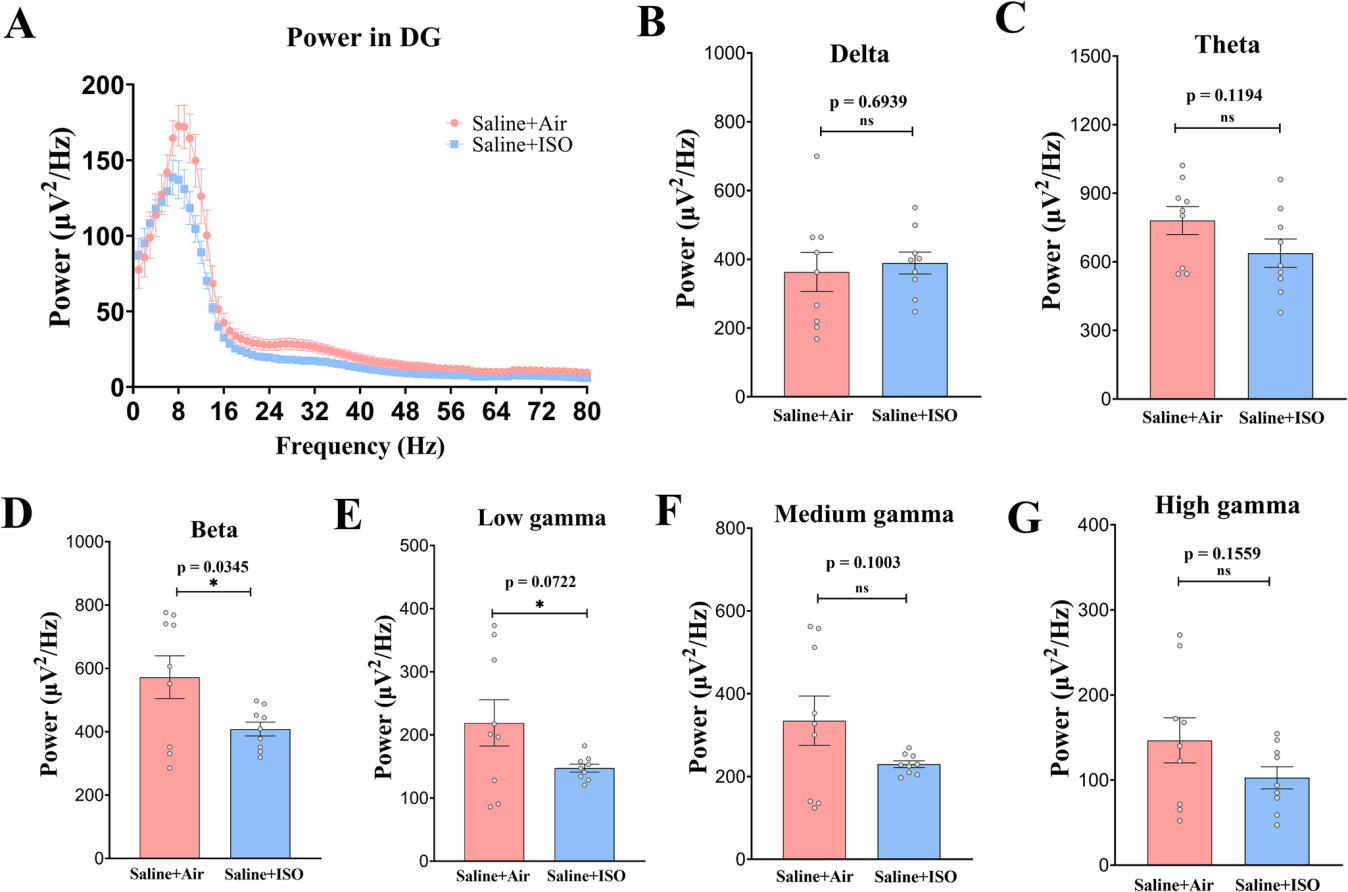
**Average power spectra of dentate gyrus** (DG) **of mice in the saline + air and saline + ISO groups differs significantly**. (A) Average power spectra obtained for local field potential (LFP) recording in the DG (three fragments/mice, three mice/group) during memory recall test. (B–G) Average power spectra in the delta (1–4 Hz; B), theta (4–12 Hz; C), medium gamma (41–65 Hz; F), and high gamma (66–80 Hz; G) bands, which indicates that there is no significant difference in the energy in the DG of hippocampus of mice in the saline + ISO group compared to the mice in the saline + air group. Average power spectra in the beta (15–25 Hz; D) and low gamma (26–40 Hz; E) bands, which implies that saline + ISO group mice have lower energy in different frequency energy spectra in DG. *p < 0.05; ***p < 0.001; ****p < 0.0001. Data are presented as mean ± SEM. ns, not significant.

In the DG, the Saline + ISO group demonstrated decreased mean power in the beta (Figure 5D, unpaired *t* test, *t*(16) = 2.311, *p* = 0.0345) compared to the Saline + Air group. In the DG, the Saline + ISO group demonstrated a decreasing trend in mean power in the low gamma (Figure 5E, unpaired *t* test, *t*(16) = 1.925, *p* = 0.0722) bands compared to the Saline + Air group.

### 3.6 Anterior Cingulate Cortex

**FIGURE 6:**
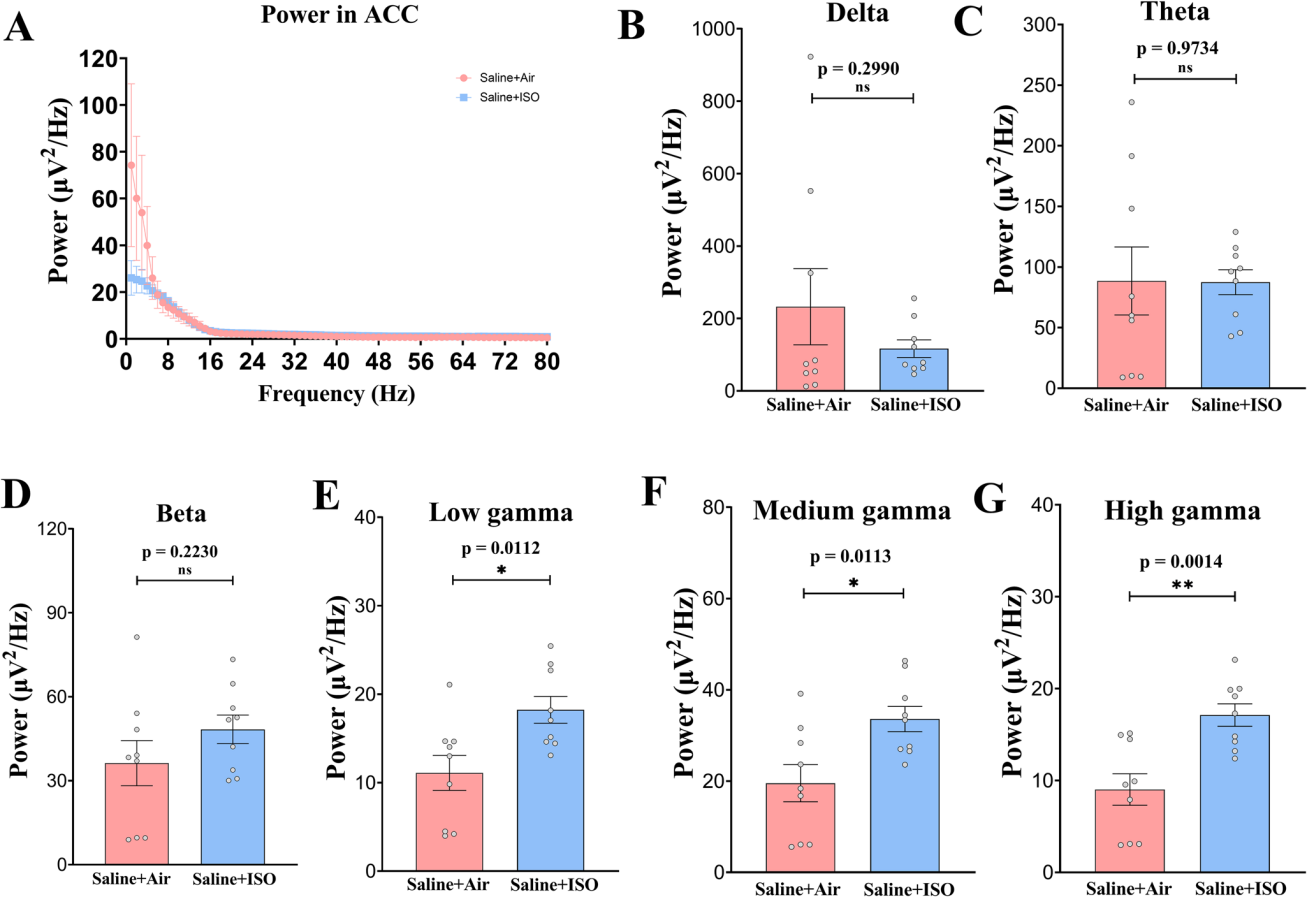
**Average power spectra of anterior cingulate cortex** (ACC) **of mice in the saline + air and saline + ISO groups differs significantly**. (A) Average power spectra obtained for local field potential (LFP) recording in the ACC (three fragments/mice, three mice/group) during memory recall test. (B–G) Average power spectra in the delta (1–4 Hz; B), theta (4–12 Hz; C), and beta (15–25 Hz; D) bands, which indicates that there is no significant difference in the energy in the ACC of hippocampus of mice in the saline + ISO group compared to the mice in the saline + air group. Average power spectra in the low gamma (26–40 Hz; E), medium gamma (41–65 Hz; F), and high gamma (66–80 Hz; G) bands, which implies that saline + ISO group mice have higher energy in different frequency energy spectra in DG. *p < 0.05; **p < 0.01. Data are presented as mean ± SEM. ns, not significant.

The ACC exhibited elevated mean power in the low gamma (Figure 6E, unpaired *t* test, *t*(16) = 2.867, *p* = 0.0112), medium gamma (Figure 6F, unpaired *t* test, *t*(16) = 2.861, *p* = 0.0113), and high gamma (Figure 6G, unpaired *t* test, *t*(16) = 3.850, *p* = 0.0014) bands in the Saline + ISO group compared to the Saline + Air group.

### 3.7 Lateral Part of the Mediodorsal Thalamus

**FIGURE 7:**
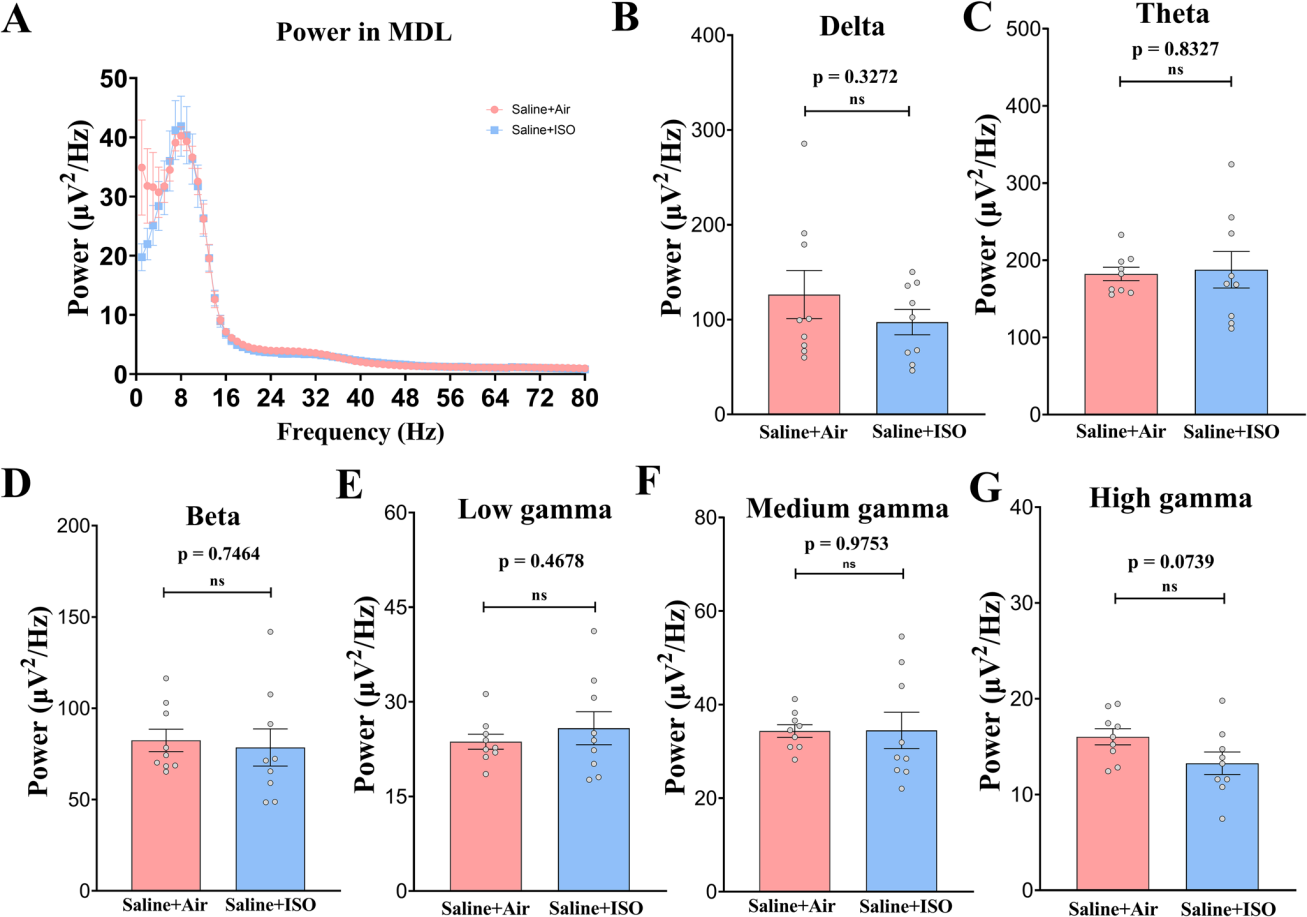
**Average power spectra in the mediodorsal thalamus of mice in the saline + air and saline + ISO groups are not significantly different**. (A) Average power spectra obtained for local field potential (LFP) recording in the mediodorsal thalamus (three fragments/mice, three mice/group) during memory recall test. (B–G) Average power spectra in the delta (1–4 Hz; B), theta (4–12 Hz; C), beta (15–25 Hz; D), low gamma (26–40 Hz; E), medium gamma (41–65 Hz; F), and high gamma (66–80 Hz; G) bands, which indicates that there is no significant difference in the energy in the mediodorsal thalamus (MDL) of hippocampus of mice in the saline + ISO group compared to the mice in the saline + air group. Data are presented as mean ± SEM. ns, not significant.

No significant differences were observed in any frequency bands of the lateral part of the MDL between the Saline + Air and Saline + ISO groups (Figure 7).

## 4 Discussion

The present study highlights those sedating male mice at P7 with ISO for 6 h results in mild cognitive impairment in adulthood. Specifically, in the benign fear memory paradigm, mice exposed to ISO exhibited enhanced fear memory. To our knowledge, similar studies are lacking in the literature, and the observed effects may be attributed to the rigor of our experimental design. Fear conditioning serves as a widely used paradigm for evaluating cognitive abilities, with freezing being a primary indicator of fear intensity. However, it is noteworthy that, under identical stimulus conditions, some experimental animals exhibit behaviors beyond freezing, such as running, jumping, or darting (Trott et al. 2022). Furthermore, we noted differential responses in female and male mice at various ages, though these data are not presented in this study. Our focus was on male adult mice aged 6–8 months. These findings underscore the potential long‐term cognitive ramifications of early‐life ISO exposure, emphasizing critical considerations for the clinical application of sedation in pediatric populations.

Mapping activated neurons proves to be an effective method for unveiling functional changes across multiple brain regions. Fear memory, residing within functionally connected engram cell ensembles, spans various brain regions, including the cerebral cortex, amygdala, thalamus, striatum, hypothalamus, and hippocampus, among others (Roy et al. 2022). Representative pathways involved in fear cognitive function encompass the “where” pathways and “freezing” pathways. The connections between the MEC and the DG are implicated in spatial navigation (Fernandez‐Ruiz et al. 2021), whereas the ACC and the MDL modulate freezing behavior (Fernandez‐Ruiz et al. 2021). In our study, we assessed neuronal activation in these pivotal brain regions upon fear memory recall 24 h post‐sedation. Surprisingly, we noted a greater number of activated neurons in the MDL in mice subjected to prolonged sedation, whereas the disparities in activation levels between the MEC, DG, and ACC were less pronounced. This observation prompts an intriguing inquiry: Why is there not a significant divergence in activation levels between the MEC, DG, and ACC? Unraveling the underlying mechanisms and network dynamics contributing to this pattern of neuronal activation could illuminate the specific effects of long‐term sedation on fear memory circuits and offer invaluable insights into the intricacies of brain function following early‐life exposure to ISO. Further exploration of the intricate interactions among these brain regions and their distinct roles in fear memory processes may elucidate the observed patterns of neuronal activation and contribute to a comprehensive understanding of the cognitive repercussions of neonatal ISO exposure.

Neuronal oscillations serve as a vital component of brain function, playing a pivotal role in all processes of learning and memory. Any perturbation in brain function is anticipated to manifest as alterations in neuronal oscillations within the pertinent brain regions. Although prior studies have predominantly focused on theta and gamma oscillations (Cardoso‐Cruz et al. 2013; Voloh et al. 2015), our investigation delves into LFP recordings in four crucial regions: the MEC, DG, ACC, and MDL. Remarkably, significant changes in gamma oscillations were noted in most brain regions of mice subjected to prolonged sedation. Notably, low gamma and medium gamma oscillations, which are particularly associated with associative memory for object encounters (Trimper et al. 2017), displayed substantial alterations. Our findings provide compelling evidence that low gamma oscillations undergo modifications across a wide spectrum of brain regions, underscoring a robust association between long‐term ISO sedation and induced cognitive changes. Elucidating the nuanced impact of ISO on gamma oscillations in diverse brain regions contributes to the expanding knowledge base concerning the intricate interplay among sedation, neuronal oscillations, and cognitive function. These results underscore the potential utility of gamma oscillations as pivotal indicators of cognitive alterations following neonatal ISO exposure.

In clinical practice, uncertainty prevails regarding the long‐term impact of ISO on cognitive function, with predominant attention focused on acute neurotoxic side effects. Our current study offers conclusive evidence that even very low doses of ISO administered over an extended period during brain development can elicit changes in adult cognitive performance. This underscores the critical importance of contemplating the potential cognitive repercussions of prolonged ISO exposure in pediatric populations. Nevertheless, the clinical landscape necessitates further research to thoroughly assess the benefits and risks associated with an exaggerated response to minor injuries. The complexities surrounding how the developing brain reacts to ISO, the underlying mechanisms of cognitive changes, and the potential long‐term ramifications warrant careful consideration. This research has the potential to inform clinical decision‐making, particularly in scenarios where sedation is imperative for medical procedures in young patients.

There are some limitations of this study. The mice we used represent only C57BL/6J mice and are not representative of other mice, such as C57BL/6N and other mice. In addition, this study only investigated the activation of four brain regions, MEC, ACC, MDL, and DG, in C57BL/6J mice, and did not consider the specific mechanisms by which exposure to low doses of ISO during juvenile development of C57BL/6J mice affects the activation of neurons in other brain regions as well as neural circuits related to fear memory. In addition, only male mice at 7 days after birth were used in this study, and the sex‐dependent effects of low‐dose ISO on the regulation of cognitive function during juvenile development in C57BL/6J mice were not considered in this study. In order to investigate the broader effects of ISO on cognitive performance, future studies should explore its effects on a wider range of disease model mice, such as the mouse model of autism spectrum disorders.

As the medical community grapples with inquiries concerning the utilization of ISO in pediatric patients, ongoing investigations should endeavor to narrow the knowledge gap, furnishing a more nuanced comprehension of the risks and benefits linked with its administration. Ultimately, such insights will aid in the formulation of guidelines and protocols that prioritize the safety and cognitive well‐being of young individuals undergoing ISO sedation.

## Author Contributions

**Jing Sun**: conceptualization, data curation, investigation, formal analysis, writing – original draft, writing – review and editing, funding acquisition. **Yong Tang**: formal analysis, investigation, writing – original draft, visualization, data curation, software, writing – review and editing. **Jinlong Chang**: funding acquisition, writing – review and editing, writing – original draft, formal analysis, visualization, investigation. **Jianbang Lin**: methodology, resources. **Li Luo**: visualization, data curation, investigation. **Yuantao Li**: investigation, data curation. **Guanxiong Wu**: data curation, investigation**. Yuting Hu**: data curation, investigation. **Zhao Zheng**: formal analysis, visualization. **Ye Zhang**: writing – review and editing, conceptualization, funding acquisition, supervision, project administration, validation.

## Conflicts of Interest

The authors declare no conflicts of interest.

## Ethics Statement

All aspects of animal husbandry and experimental procedures were conducted in accordance with ethical standards and all procedures involving mice were approved by the Animal Care and Use Committee of Shenzhen Institute of Advanced Technology (SIAT), Chinese Academy of Sciences (CAS) (Approval No. YSB‐20220512‐SJ‐A0555) and complied with the ARRIVE guidelines 2.0.

## Peer Review

The peer review history for this article is available at https://publons.com/publon/10.1002/brb3.70816

## Data Availability

The data that support the findings of this study are available from the corresponding author upon reasonable request. Some data may not be made available because of privacy or ethical restrictions.
